# The Intersection of Mental Health, Self-Reported Neurocognitive Functioning, and Education in Older HIV+ Gay Men

**DOI:** 10.1093/geroni/igaa057.2563

**Published:** 2020-12-16

**Authors:** Kristen Krause

**Affiliations:** Rutgers University, Piscataway, New Jersey, United States

## Abstract

Older people living with HIV/AIDS (PLWHA) face different mental and neurocognitive challenges related to their health and well-being. Using data from a cross-sectional study (n=250) on older (age 50-69) gay men living with HIV/AIDS in NYC, this study examined the multi-level associations between self-reported neurocognitive functioning, mental health, and key sociodemographics (age, race/ethnicity, financial situation, and education). Findings suggest those who have higher self-reported neurocognitive functioning have higher levels of education, better self-rated health, and lower levels of PTSD and depression (p<0.01). Differences were not observed based on race/ethnicity, financial situation, and age. The overall findings demonstrate educational differences in self-reported cognitive functioning among older HIV+ gay men and highlight the importance of enhancing interventions and policies to promote better cognitive and mental health outcomes. More research is warranted to understand the intersection of education and cognitive performance among other sub-groups of PLWHA to understand whether these findings are consistent.

